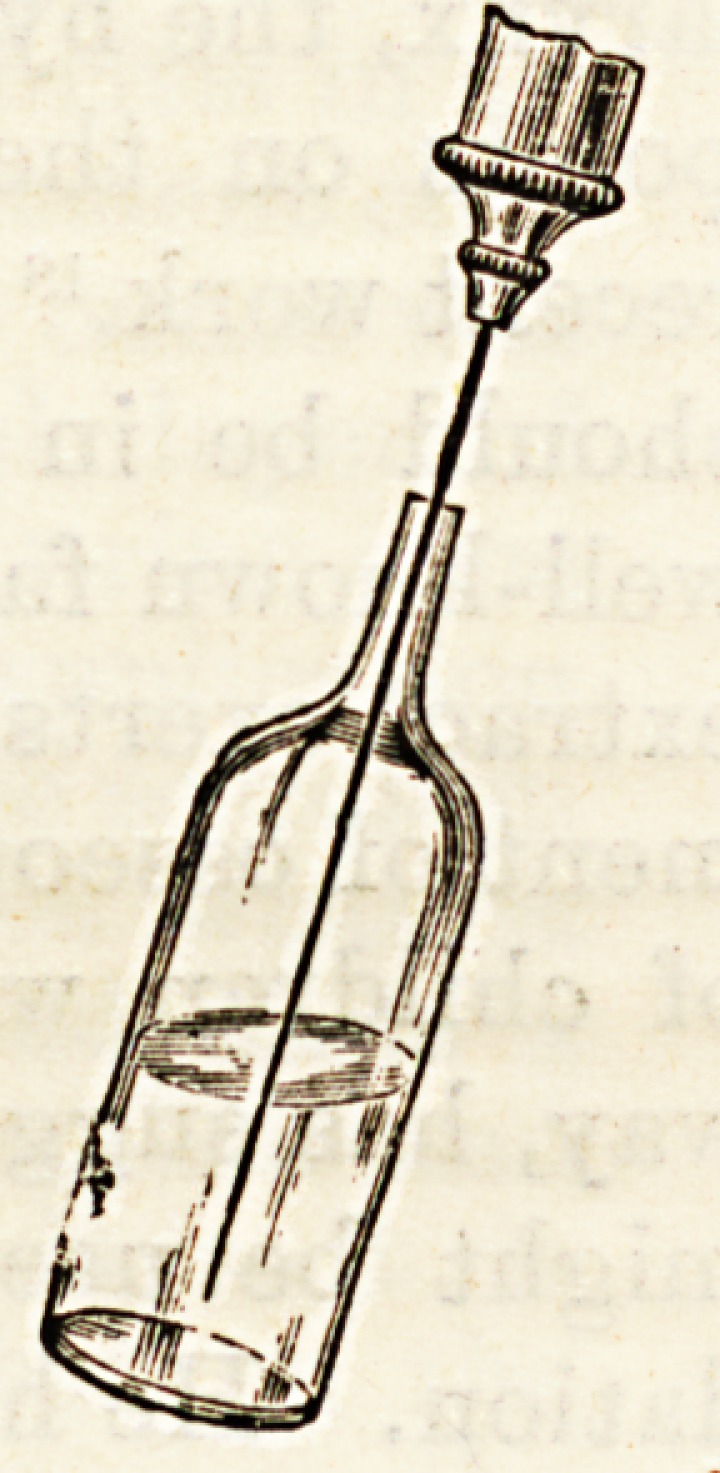# New Appliances and Things Medical

**Published:** 1897-12-25

**Authors:** 


### NEW APPLIANCES AND THINGS MEDICAL.

[We shall be glad to receive, at our Office, 28 & 29, Southampton Street, Strand, London, W.C., from the manufacturers, specimens of all new preparations and appliances which may be brought out from time to time.]

### BROWN BREAD FOR DIABETICS.

(Callard and Co., 65, Regent Street.)

We have received samples of this new bread from Callard and Co. In appearance and taste it closely resembles ordinary brown bread, though perhaps a little heavier and more filling than the genuine article. Considering, however, that this result is said to be obtained without the addition of any farinaceous or carbohydrate ingredient, it is a truly wonderful achievement. It will be a welcome addition to usually monotonous diabetic dietary.

### HYPODERMULES.

(Frank A. Rogers, 327, Oxford Street, W.)

The above name has been applied by Mr. Rogers to an ingenious contrivance for supplying accurate quantities of sterilised hypodermic solutions. Each hypodermic dose is supplied in a small glass flask or "hypodermule," the end of which is sealed by fusion in a flame. Among the many advantages of this method may be mentioned (1) absolute sterility, (2) accurate dosage, (3) saving of time. For animal serums this method is particularly applicable.

### AUTOMATIC DISINFECTANT JAR.

(Vigor and Co., 21, Baker Street, London, W.)

We have received a sample of the above disinfectant jar, which is distinguished as the "X L." It is a cylindrical receptacle containing permanganate of potash crystals and closed at both ends. Water, however, can percolate through the porcelain, of which it is composed, dissolve the antiseptic contents, and again diffuse out into the surrounding water. It is intended for the automatic disinfection of the water in cisterns, flushing-out tanks, and the like. As a safeguard against infection from these causes, and as a powerful deodorant there can be no two opinions about this contrivance. The objection to its universal adoption, however, consists in the following points: Permanganate of potash in solution is not a practicable disinfectant for a closet, firstly, because it stains the pans, and, secondly. because it oxidises too readily the metallic fittings of the cistern, and the pipes themselves if they be of iron. The ingenuity of the firm who are introducing this novelty in disinfection will doubtless overcome these objections.

### MINCASEA.

(Thomas Barron-Brooke, 20, Cheapside, London, E.C.)

This is an artificial food for infants prepared by the above firm; when added to diluted milk, according to the directions, the resultant is closely analogous to human milk. For infants that are reared by hand this preparation has many advantages, and will be tolerated by many infantile stomachs that reject the more usual foods. It should, however be administered under medical direction, for the table of the quantities to be given at different ages, as supplied by the manufacturers with each packet, would be most unsuitable in many cases if followed out to the letter. For instance, during the first month, at any rate, during its first moiety, four ounces of food is a larger quantity than can be tolerated by the infant. With this reservation we can safely recommend "Mincasea" to those who have the charge of infants, for use in cases where milk by itself disagrees.

### SOLUBLE COCOA.

(C. J. Van Houten and Zoon, Weesp, Holland.)

This cocoa is to be highly recommended for invalid use. It contains all the nourishing and stimulating properties of the raw product, without that excess of fat in its unaltered condition which renders many otherwise excellent cocoas intolerable to the delicate stomach of invalids. Cocoa is often regarded as a bilious condiment or food, but cocoa essence such as that of Van Houten is as innocent of this disagreeable property as an ordinary infusion of tea. Van Houten's Cocoa is highly concentrated, highly soluble, and highly to be recommended for invalids.

## Figures and Tables

**Figure f1:**